# The microtubule network enables Src kinase interaction with the Na,K-ATPase to generate Ca^2+^ flashes in smooth muscle cells

**DOI:** 10.3389/fphys.2022.1007340

**Published:** 2022-09-23

**Authors:** Salomé Rognant, Violetta V. Kravtsova, Elena V. Bouzinova, Elizaveta V. Melnikova, Igor I. Krivoi, Sandrine V. Pierre, Christian Aalkjaer, Thomas A. Jepps, Vladimir V. Matchkov

**Affiliations:** ^1^ Department of Biomedical Sciences, University of Copenhagen, Copenhagen, Denmark; ^2^ Department of General Physiology, St. Petersburg State University, St. Petersburg, Russia; ^3^ Department of Biomedicine, Aarhus University, Aarhus, Denmark; ^4^ Marshall Institute for Interdisciplinary Research, Marshall University, Huntington, WV, United States

**Keywords:** Na,K-ATPase, Src kinase, intracellular Ca^2+^ signaling, microtubule network, Ca^2+^ flashes

## Abstract

**Background:** Several local Ca^2+^ events are characterized in smooth muscle cells. We have previously shown that an inhibitor of the Na,K-ATPase, ouabain induces spatially restricted intracellular Ca^2+^ transients near the plasma membrane, and suggested the importance of this signaling for regulation of intercellular coupling and smooth muscle cell contraction. The mechanism behind these Na,K-ATPase-dependent “Ca^2+^ flashes” remains to be elucidated. In addition to its conventional ion transport function, the Na,K-ATPase is proposed to contribute to intracellular pathways, including Src kinase activation. The microtubule network is important for intracellular signaling, but its role in the Na,K-ATPase-Src kinase interaction is not known. We hypothesized the microtubule network was responsible for maintaining the Na,K-ATPase-Src kinase interaction, which enables Ca^2+^ flashes.

**Methods:** We characterized Ca^2+^ flashes in cultured smooth muscle cells, A7r5, and freshly isolated smooth muscle cells from rat mesenteric artery. Cells were loaded with Ca^2+^-sensitive fluorescent dyes, Calcium Green-1/AM and Fura Red/AM, for ratiometric measurements of intracellular Ca^2+^. The Na,K-ATPase α2 isoform was knocked down with siRNA and the microtubule network was disrupted with nocodazole. An involvement of the Src signaling was tested pharmacologically and with Western blot. Protein interactions were validated with proximity ligation assays.

**Results:** The Ca^2+^ flashes were induced by micromolar concentrations of ouabain. Knockdown of the α2 isoform Na,K-ATPase abolished Ca^2+^ flashes, as did inhibition of tyrosine phosphorylation with genistein and PP2, and the inhibitor of the Na,K-ATPase-dependent Src activation, pNaKtide. Ouabain-induced Ca^2+^ flashes were associated with Src kinase activation by phosphorylation. The α2 isoform Na,K-ATPase and Src kinase colocalized in the cells. Disruption of microtubule with nocodazole inhibited Ca^2+^ flashes, reduced Na,K-ATPase/Src interaction and Src activation.

**Conclusion:** We demonstrate that the Na,K-ATPase-dependent Ca^2+^ flashes in smooth muscle cells require an interaction between the α2 isoform Na, K-ATPase and Src kinase, which is maintained by the microtubule network.

## Introduction

The Na,K-ATPase extrudes three Na^+^ ions from the cell in exchange for two K^+^ ions, powered by the hydrolysis of one ATP molecule (Blanco and Mercer, 1998). This ion transport is essential for maintaining the transmembrane ion gradient, which is important for the cell membrane potential and multiple secondary active transports. The Na,K-ATPase is the heterodimeric plasma membrane protein that consists of, at least, α- and β-subunits (Geering, 2008). The α subunit is responsible for ion translocation and catalytic activities of the enzyme (Blanco et al., 1994). It contains the binding sites for ATP and specific inhibitors of the Na,K-ATPase, cardiotonic steroids. Four isoforms of the α subunit have been identified (Blanco and Mercer, 1998) and their tissue-specific expression profile has been described (Matchkov and Krivoi, 2016). Vascular smooth muscle cells co-express both the α1 and α2 isoforms of Na,K-ATPase but they have been proposed to serve different functions (Matchkov, 2010; Blaustein et al., 2016). Importantly, while the housekeeping α1 isoform Na,K-ATPase is homogeneously distributed over cell membrane and nearly saturated at resting conditions, the α2 isoform forms membrane microdomains and is involved in numerous signaling functions (Matchkov, 2010; Zhang et al., 2019; Blaustein and Hamlyn, 2020). In rodent tissues, the α1 and α2 isoforms of Na,K-ATPase can be differentiated pharmacologically because of their different affinity to cardiotonic steroid, ouabain, i.e., the α2 isoform is approximately 100-fold more sensitive to ouabain than the α1 isoform (Blanco and Mercer, 1998). That is, the rodent α2 isoform can be inhibited by sub-micromolar and micromolar concentrations of ouabain, while 500 μM–1 mM ouabain is needed to inhibit the α1 isoform Na,K-ATPase.

The Na,K-ATPase was also proposed as a plasma membrane receptor that initiates intracellular signaling pathways upon binding with some cardiotonic steroids, including ouabain (Aizman and Aperia, 2003; Aperia, 2007). It has been shown that binding of ouabain to the Na,K-ATPase leads to Src kinase auto-phosphorylation and, thus, its activation (Haas et al., 2002), which initiates several downstream intracellular signaling pathways (Xie, 2003). Although the mechanism of this Na,K-ATPase-dependent Src kinase activation remains debated (Li and Xie, 2009; Weigand et al., 2012; Yosef et al., 2016; Cui and Xie, 2017), its consequences for vascular functions were reported (Zulian et al., 2013; Hangaard et al., 2017; Bouzinova et al., 2018; Staehr et al., 2018; Zhang et al., 2018). In the vascular wall, micromolar concentrations of ouabain inhibit only the α2 isoform Na,K-ATPase and have no significant effect on the global intracellular Ca^2+^ in smooth muscle cells (Mulvany et al., 1984; Aalkjaer and Mulvany, 1985; Matchkov et al., 2007; Matchkov et al., 2012). However, these concentrations of ouabain also potentiate vascular tone and vasocontraction (Mulvany et al., 1982; Aalkjaer and Mulvany, 1985; Matchkov et al., 2007), and increase blood pressure *in vivo* (Yuan et al., 1993; Zhang et al., 2005). We have recently demonstrated that these effects of ouabain mediate via Src kinase signaling in the vascular wall (Hangaard et al., 2017; Bouzinova et al., 2018; Staehr et al., 2018).

In previous studies, micromolar concentrations of ouabain did not affect the global intracellular Ca^2+^ concentration (Mulvany et al., 1984; Aalkjaer and Mulvany, 1985; Matchkov et al., 2007; Matchkov et al., 2012), but did induce spatially restricted Ca^2+^ transients in cultured aortic smooth muscle cells and in rat mesenteric artery wall (Matchkov et al., 2007). These Ca^2+^ transients are suggested to be important to control intercellular coupling and vascular tone (Matchkov et al., 2007; Matchkov et al., 2012), but the underlying mechanism is unknown.

Microtubules are an integral component of the cytoskeleton, which mediates many cellular functions, including intracellular signaling (Jepps, 2021). It has been shown that Na,K-ATPase activity can be modulated by microtubules and might serve as an anchorage site at the plasma membrane (Alonso et al., 1998; Casale et al., 2003; Arce et al., 2008). Although the role of the microtubule network in modulation of Src kinase signaling was proposed (Abu-Amer et al., 1997; Yamada et al., 2000; Wu et al., 2008), its importance in this pathway in smooth muscle cells is still unknown. Moreover, whether the microtubule network plays an important role in Na,K-ATPase-dependent Src kinase signaling (Cui and Xie, 2017) remains to be elucidated.

In this study, we aimed to characterize the mechanism behind spatially restricted sub-membrane Ca^2+^ transients induced in smooth muscle cells by micromolar concentrations of ouabain. Specifically, we have questioned whether these Ca^2+^ transients are a direct product of local ion homeostasis disbalance because of ion transport inhibition (Blaustein and Lederer, 1999) or a result of the Na,K-ATPase-dependent intracellular signal transduction (Cui and Xie, 2017). We also questioned here the importance of the microtubule network for generation of Ca^2+^ transients and its interplay with potential signalling pathways. We have addressed these research questions in cultured aortic smooth muscle cells, A7r5 that were originally used to characterize the ouabain-induced Ca^2+^ transients (Matchkov et al., 2007), and validated our findings in vascular smooth muscle cells isolated from rat mesenteric arteries. We studied the changes in Ca^2+^ transients produced by a modulation of ion transport by the Na,K-ATPase, by pharmacological intervention with tyrosine phosphorylation and by the α2 isoform Na,K-ATPase downregulation. Involvement of signal transduction was validated with proximity ligation assay and phosphoprotein-specific Western blot analyses. The functional role of microtubule network was studied with pharmacological disruption by nocodazole.

## Methods

### Intracellular Ca^2+^ imaging in cultured rat aortic smooth muscle cells—A7r5

*Mycoplasma* free A7r5 (American Type Culture Collection - ATCC; VA, United States) were cultured in DMEM medium (*In Vitro*, Denmark) supplemented with 10% fetal calf serum, 1% L-glutamine and 0.1% KPS (kanamycin 2 g, penicillin 1 million IU, streptomycin 1 g in 20 ml phosphate-buffered saline [PBS, in mM: NaCl 137, KCl 2.7, Na_2_HPO_4_ 8.2, KH_2_PO_4_ 1.8, at pH 7.4)). Confluent A7r5 cells were detached by nonenzymatic cell dissociation solution (Sigma-Aldrich, Denmark), resuspended in DMEM medium as above and pipetted into 200 µl customer-made imaging chambers with cover glass bottom. The cells were stored for 3 h in humidified 37°C/5% CO_2_ cell incubator to allow the cells to attach to the bottom prior the experiment imaging experiment.

To measure changes of intracellular Ca^2+^ transients, the cells were loaded with a mixture of Calcium Green-1/AM (3 μM) and Fura Red/AM (6 μM) for 15 min at 37°C/5% CO_2_. Both dyes were initially dissolved in DMSO maintaining final concentration ≤0.01%. Calcium Green-1/AM and Fura Red/AM mixture was initially prepared in physiological salt solution (PSS, in mM: 119 NaCl, 3.0 KCl 1.18 KH_2_PO_4_, 1.17 MgCl_2_, 25.0 NaHCO_3_, 0.026 EDTA and 5.5 glucose, gassed with 5% CO_2_ in air and adjusted to pH 7.4) and this solution then replaced the cell medium in the imaging chamber.

Prior the imaging protocol, the cells were washed five times with warmed to 37°C PSS that was gassed with 5% CO_2_ in air. The experiments were performed using an inverted confocal laser scanning microscope (LSM 5 Pa Exciter or LSM780, Zeiss GmbH, Germany) and images were acquired with a water immersion objective (×63, 1.2 W, Zeiss GmbH, Germany).

Both Fura Red and Calcium Green-1 were excited at 488 nm, the emission from dyes was collected above 560 nm and in the interval from 505 to 530 nm, respectively. The elevation in intracellular Ca^2+^ resulted in increased fluorescence intensity of Calcium Green-1 and decreased fluorescence intensity of Fura Red. Therefore, a combination of these two Ca^2+^ indicators was used for ratiometric assessment of intracellular Ca^2+^ changes, as described previously (Matchkov et al., 2007).

PSS in the imaging chamber was kept at 37°C by thermostatically controlled chamber holder and constantly gassed with 5% CO_2_ in air. The experimental protocol consisted of 5 min baseline recordings, and the pharmacological intervention by replacement of the solution in the chamber to another modified solution and followed by incubation for 15 min prior next 5 min imaging session. Up to three pharmacological interventions per single experimental protocol were done.

Changes in intracellular Ca^2+^ were analyzed using either the Zeiss LSM Image Examiner program or ZEN software (Zeiss GmbH, Germany). The local Ca^2+^ events were analyzed as a profile of fluorescence ratio (Calcium Green-1 over Fura Red) either at cell perimeter or in the center of the cell, and plotted for the profile distance, as described previously (Matchkov et al., 2007). Ca^2+^ transients were counted if elevation of intracellular Ca^2+^ was 5 or more times above averaged baseline. The number of Ca^2+^ transients was normalized to the length of profile the fluorescence was measured. The analysis was done blind with custom-made Makro in Excel Spreadsheet Software (Microsoft corp., NM, United States).

### siRNA transfection of A7r5 cells

Transfection of A7r5 cells was done as described previously (Matchkov et al., 2008). The siRNA directed against the α2 isoform (Ambion; the sense sequence: 5′-GAG​AAC​ATC​TCC​GTG​TCA​tt-3′) and another control, nonrelated siRNA directed against enhanced green fluorescent protein (Ambion; the sense sequence: 5′-CCA​CUA​CCU​GAG​CAC​CCA​Gtt-3′) were used as previously described (Matchkov et al., 2012). When A7r5 cells were 80% confluent, antibiotics were omitted from DMEM medium for 2 h. The cells were then transfected by TransIT TKO transfection kit (Mirus Bio Co., United States) following the protocol given by the manufacturer. Each culture dish was transfected with 5 μl TKO and 25 nmol/L siRNA in antibiotic-free DMEM medium for 8 h, then the cells were washed out and the medium was replaced back to standard DMEM. The cells were used 72 h later for functional and expressional analyses.

### Semi-quantification of the Na,K-ATPase expression

Confluent A7r5 cells were washed in the culture dishes (Falcon, Becton Dickson, Denmark) 3 times in ice-cold PBS solution and collected by scrubbing. The cell suspension was centrifuged at 3,000-g and collected pellet was homogenized in lysis buffer (in mM: Tris‐HCl 10, sucrose 250, EDTA 1, EGTA 1; Triton X‐100 2%, pH 7.4; 1 tablet protease inhibitor per 10 ml and 0.01 mmol−1 of Halt’s phosphatase inhibitor cocktail) and 2x trisglycine sodium dodecyl sulphate (SDS) sample buffer (Invitrogen, MA, United States) with 1 mol/L DTT. The homogenates were ultrasonicated for 45 s and centrifuged at 10,000-g for 10 min at 4°C. The supernatants were collected, and the protein contents were determined using a bicinchoninic acid protein assay reagent kit (Pierce, United States). The sample was adjusted then with 1 M DTT and 2x Tris-glycine SDS sample buffer (Invitrogen, MA, United States) with an approximate ratio of 10:3:3.

Proteins (10 µg) were separated on 14% Tris-glycine gels and electrotransferred onto nitrocellulose membranes, which were then blocked by incubation in 5% nonfat dry milk in PBS with 0.5% vol/vol Tween 20 (PBS-T). The membrane was divided at approximately 64 kDa and the upper part of the membrane was incubated with primary α2 isoform Na,K-ATPase antibody (1:2,000; Millipore, United States [Catalog no. AB9094]) overnight at 5°C in PBS-T. The lower part of the membrane was incubated with pan-actin antibody (1:2,000, Cell Signaling Technology Inc., United States [Catalog no.4968]). Next day, the membranes were washed and incubated with horseradish peroxidase-conjugated secondary antibody (Dako, Denmark) for 1 h and bound antibody was detected by an ECL chemiluminescence kit (Amersham, United Kingdom).

Total protein lysates from rat mesenteric arteries were homogenized in 200 μl of RIPA buffer (in mM): 50 Tris pH 8.5, 150 NaCl, 1% SDS, 1% Nonidet P40, 0.5% Sodium deoxycholate and protease inhibitor cocktail (Roche, Switzerland) for 10 min at 4°C. The supernatant was collected after centrifugation at 11,000-g for 10 min at 4°C followed by protein quantitation using a bicinchoninic acid (BCA) Protein Assay kit (Thermo Fisher Scientific, MA, United States). 20 µg of protein were loaded on SDS-PAGE gels (4%–12% bis–tris; Invitrogen, MA, United States), subjected to electrophoresis, and then transferred onto a polyvinylidene fluoride (PVDF) membrane (Immobilon®-FL, Sigma-Aldrich, Denmark). The membrane was probed with antibodies against Na,K-ATPase α2 isoform (1:2,000; Millipore, United States [Catalog no. AB9094]) and GAPDH (1:10,000; Abcam, United Kingdom [Catalog no.ab181602]). Fluorescently conjugated secondary antibodies (1:10,000; Li-Cor Biosciences [Catalog no.926-32211 and 926-32210]) were used to visualize protein bands. The membrane was imaged and analysed on the Odyssey Infrared Imaging System (Li-Cor Biosciences; version 5.2.5).

The protein expression was semi-quantified as a ratio of intensity of bands for the α2 isoform Na,K-ATPase and either pan-actin or GAPDH proteins. Analysis was done with the ImageJ (National Institutes of Health, MD, United States). The normalized protein ratio is shown in percentage of mean ratio in the control group.

### Semi-quantification of Src kinase signaling

A7r5 cells were lysed in lysis buffer as above. Ten µg of protein in Laemmli sample buffer (Bio‐Rad, Hercules, CA) was loaded onto 4–20% precast polyacrylamide stain‐free gels (Criterion TGX Stain‐free precast gel; Bio‐Rad). Total protein load was detected on the stain‐free gels using UV light in imaging system (c600; Azur Biosystems, Dublin, CA). The proteins were electrotransferred onto nitrocellulose membranes that were blocked in 3% bovine serum albumin (BSA) in Tris‐buffered solution (TBS, mmol L−1: 10 Tris‐HCl, 100 NaCl; pH 7.6) with 0.5% Tween-20 (TBST) to identify the phosphorylated Src or in 0.3% iBlock in TBS to identify total Src kinase. Membranes were incubated overnight at 4°C with primary antibodies against phosphorylated pY416 Src (1:500; Cell Signaling Technology Inc., MA, United States [Catalog no. CST-6943]) or total Src (1:200; Santa Cruz Biotechnology, TX, United States [Catalog no. sc-8056]). Next day, the membranes were incubated with horseradish peroxidase (HRP) conjugated secondary antibody (1:2000; Dako, Denmark) for 2 h at room temperature, and bound antibodies were detected by an enhanced chemiluminescence kit (ECL, Amersham, United Kingdom). Analysis was done with ImageJ (National Institutes of Health, MD, United States). Detected protein was normalized as a ratio to total protein load measured in the membrane for the same sample and expressed either as percentage of mean ratio in the control group or as a relative level of phosphorylated Src form over the total expression level of Src kinase.

### Proximity ligation assay

PLA technique was used to determine the co-localization of the α2 isoform Na,K-ATPase with Src kinase in cultured A7r5 cells and freshly isolated rat mesenteric artery myocytes. Duolink *in situ* PLA detection kit 563 (Sigma-Aldrich, Denmark) was used in accordance with the manufacturer’s instructions. Cell isolation from third-order rat mesenteric arteries was conducted as described previously (Jepps et al., 2015). Briefly, cells were fixed in 4% paraformaldehyde in PBS for 20 min and permeabilized in PBS containing 0.1% of Triton X-100 for 5 min. Cells were incubated for 1 h at 37°C in Duolink blocking solution to avoid any unspecific binding. The primary antibodies used against the α2 isoform Na,K-ATPase (1:100; Millipore, United States [Catalog no. 07-674]) and Src (1:100; Santa Cruz Biotechnology, TX, United States [Catalog no. sc-8056]) were diluted in Duolink blocking solution and incubated overnight at 4°C. Combinations of secondary anti-rabbit or anti-mouse antibodies of PLA PLUS and MINUS probes were used followed by hybridization, ligation and amplification steps. Red punctae representing proteins that are located within 40 nm of each other were visualized and quantified using a Zeiss LSM710 upright laser scanning confocal microscope.

### Animals

All animal experiments were performed in accordance with Directive 2010/63EU on the protection of animals used for scientific purposes and approved by the national ethics committee, Denmark. Male Wistar rats were purchased from Janvier Labs (France), group-housed in clear plastic containers and underwent at least 1 week of habituation before use. All experiments with dissected mesenteric arteries were performed using 12- to 15-week-old male Wistar rats.

### Reagents

Calcium Green-1/AM and Fura Red/AM were obtained from Invitrogen (MA, United States). Nocodazole was obtained from Tocris (United Kingdom). The pNaKtide peptide was obtained from HD Biosciences (China) and stock solution was prepared in water (10 mM) and stored at −20°C. All other chemicals were purchased from Sigma-Aldrich (Denmark). Stock solutions of nocodazole, genistein and PP2 were prepared in DMSO (10 mM) and stored at −20°C. Ouabain stock solution was prepared on the day of experiment (minimum 2 h prior to application) in a concentration of mM in water. Drugs were applied a minimum 15 min prior to measurements/interventions.

### Statistical analysis

All statistical analysis was performed using GraphPad Prism 8 (GraphPad Software Inc., San Diego, CA, United States). Different statistical tests were performed throughout the present study, as appropriate, and are defined in the Results section for each data set analysed. The data were subjected to a normality test (Shapiro-Wilk test) that defines the use of either parametric or nonparametric statistical analysis. All data are presented as means ± standard error of the mean (SEM). A probability (*P*) level of <0.05 was considered significant, and n refers to number of experiments in case of cell culture. The number of culture dishes used at the day of experiment is also indicated. When mesenteric arteries were studied, n refers to number of cells and the number of rats is also provided.

## Results

### Ouabain-induced Ca^2+^ flashes depend on the expression of Na,K-ATPase α2 isoform

**FIGURE 1:**
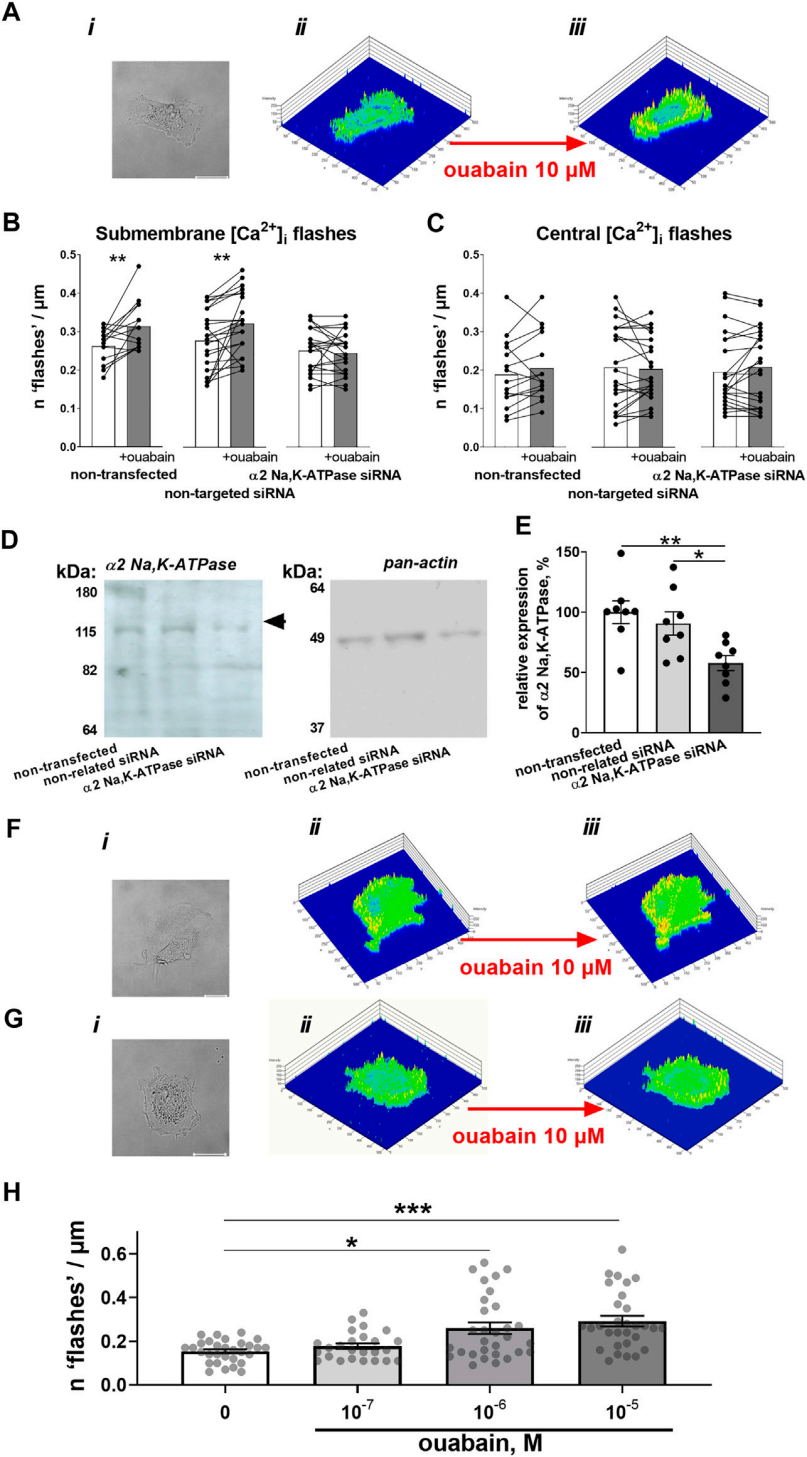
Ouabain induces Ca^2+^ flashes that depend on the expression of the α2 isoform Na,K-ATPase. A7r5 cells (**A** *i*) were loaded with Ca^2+^ sensitive dyes, Calcium Green-1 and Fura Red, which ratio represents relative intracellular Ca^2+^ changes. This ratio was assessed under control conditions (**A** *ii*) and after incubation with 10 µM ouabain (**A** *iii*). Exposure to ouabain induced significant elevation of submembrane Ca^2+^ flashes in non-transfected cells (*n* = 15 of 3 cultures) and cells transfected with non-targeted siRNA (*n* = 25 of 3 cultures), while cells transfected with siRNA directed against α2 isoform Na,K-ATPase did not show any changes (*n* = 22 of 3 cultures) **(B)**. No significant changes of intracellular Ca^2+^ activity in the center of cell was seen in any of the groups **(C)**. The transfection with siRNA directed against α2 isoform Na,K-ATPase but not with non-targeted siRNA reduced the expression of α2 isoform Na,K-ATPase (*n* = 8) [**(D)**—representative Western blot, **(E)** averaged results]. Representative cell images (*i*), and Calcium Green-1/Fura Red intensity ratio under control conditions (*ii*) and in the presence of ouabain (*iii*) for A7r5 cells transfected with non-targeted siRNA **(F)** and siRNA directed against the α2 isoform Na,K-ATPase **(G)**. The effect of ouabain on the Ca2+ flashes was concentration-dependent [**(H)**; *n* = 25–30 of 9 cultures]. *, ** and ***, *p* < 0.05, 0.01 and 0.001 (two-way ANOVA followed by Sidak’s multiple comparisons test in B and C; one-way ANOVA followed by Tukey’s multiple comparisons test in E and Kruskal–Wallis’ test followed by Dunn’s multiple comparison test in **(H)**.

In A7r5 cells, superfusion with 10 µM ouabain induced spatially restricted [Ca^2+^]_i_ transients—“[Ca^2+^]_i_ flashes” (Figure 1A). Similar to a previous report (Matchkov et al., 2007), these Ca^2+^ flashes were only detected on the cell periphery (Figure 1B), while no ouabain-induced Ca^2+^ fluctuations were seen in the center of the cells (Figure 1C). Transfection of A7r5 cell with siRNA directed against the α_2_ isoform suppressed the protein expression of the α_2_ isoform Na,K-ATPase compared to cells transfected with non-targeted siRNA (Figure 1D&E). The cells transfected with non-targeted siRNA were still able to increase submembrane Ca^2+^ flashes upon administration of ouabain (Figure 1F). In contrast, A7r5 cells transfected with siRNA targeted against the Na,K-ATPase α_2_ isoform did not show any ouabain-induced Ca^2+^ flashes (Figure 1G). The effect of ouabain was concentration dependent. Ca^2+^ flashes were induced by ouabain at 10^−6^ and 10^−5^ M while no significant effect of 10^−7^ M was seen (Figure 1H).

### The Na,K-ATPase ion transport is not essential for Ca^2+^ flashes

**FIGURE 2:**
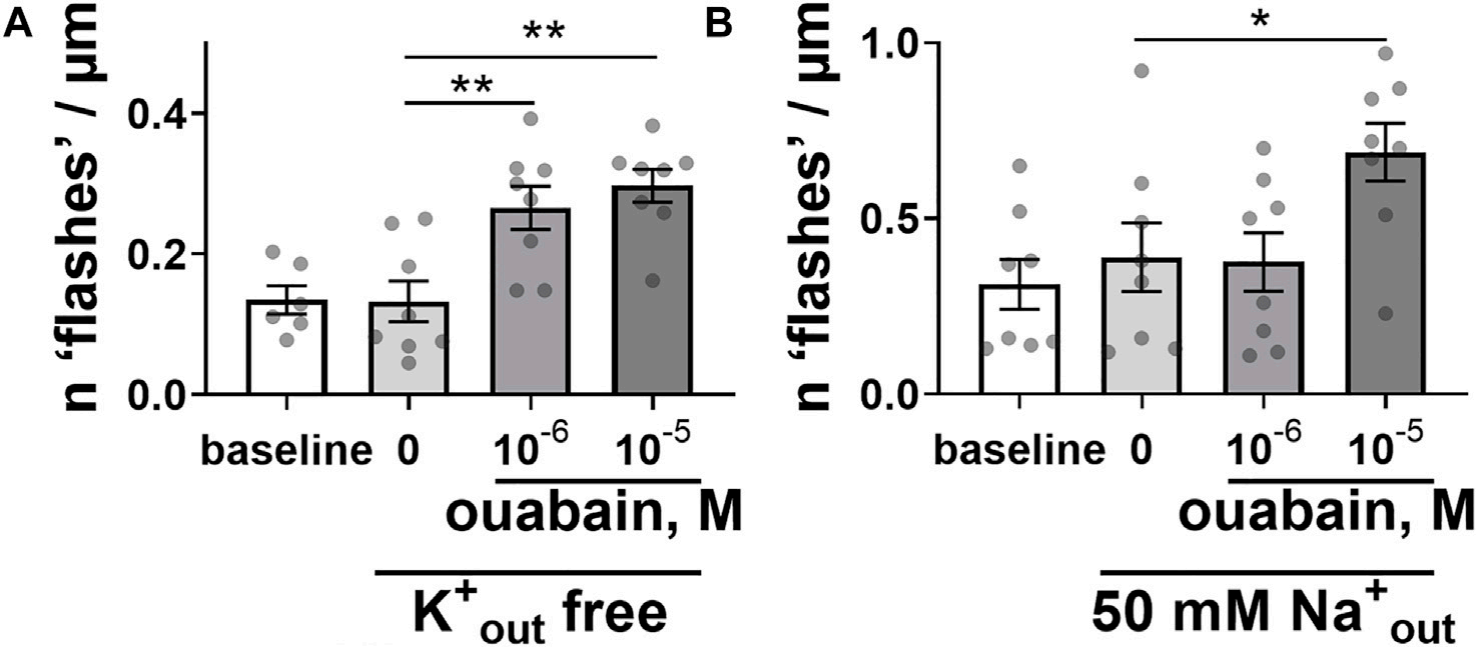
Modulation of the Na,K-ATPase ion transport activity did not prevent the ability of ouabain to induce intracellular Ca^2+^ flashes. Ouabain induced Ca^2+^ flashes after omission of K^+^ ions from extracellular solution [**(A)**; *n* = 6–8 of 5 cultures], and a reduction of extracellular Na^+^ concentration to 50 mM [**(B)**; *n* = 8 of 6 cultures]. * and **, *p* < 0.05 and 0.01 (one-way ANOVA followed by Sidak’s multiple comparisons test).

We have tested the possibility that Ca^2+^ flashes originated from changes in activity of the Na^+^,Ca^2+^-exchanger, because of inhibition of Na^+^ extrusion by the Na,K-ATPase with ouabain (Blaustein and Lederer, 1999). When the Na,K-ATPase ion transport was modulated by changes in the extracellular concentrations of transported K^+^ and Na^+^ cations, the ability of ouabain to elicit the intracellular Ca^2+^ flashes was still preserved (Figure 2). Omission of extracellular K^+^ suppresses the ion pumping by Na,K-ATPase (Holmgren et al., 2000). However, ouabain-induced Ca^2+^ flashes were not affected (Figure 2A). Reduction of extracellular Na^+^ to 50 mM, which is expected to potentiate the Na,K-ATPase ion transport, reduced the potency of ouabain to induce the intracellular Ca^2+^ flashes but did not prevent them (Figure 2B).

### Activation of the Ca^2+^ flashes by ouabain requires Src kinase activation by phosphorylation

**FIGURE 3:**
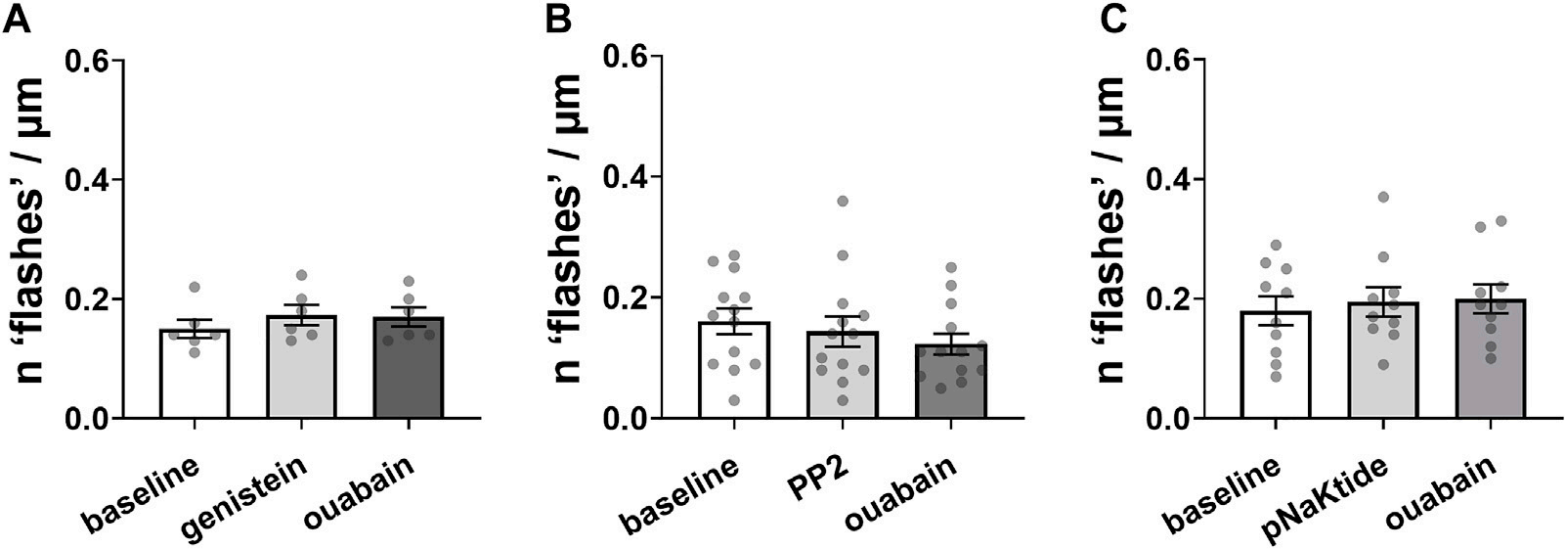
Ouabain-induced Ca^2+^ flashes depend on Src kinase activation by phosphorylation. Inhibition of tyrosine phosphorylation by 50 µM genistein [**(A)**; *n* = 6 of 3 cultures] or inhibition Src kinase activation with 10 µM PP2 [**(B)**; *n* = 13 of 7 cultures] prevented Ca^2+^ flashes induced by 10 µM ouabain. Ca^2+^ flashes were also not seen in the presence of a specific inhibitor of the Na,K-ATPase-dependent Src kinase activation, pNaKtide (1 µM) [**(C)**; *n* = 10 of 6 cultures]. The data are compared with a one-way ANOVA followed by Sidak’s multiple comparisons test.

Micromolar concentrations of ouabain are known to induce auto-phosphorylation and activation of non-receptor tyrosine kinase, Src (Hangaard et al., 2017). We tested the involvement of this pathway in generation of ouabain-induced Ca^2+^ flashes. An unspecific tyrosine kinase inhibitor, genistein prevented ouabain-induced Ca^2+^ flashes in A7r5 cells (Figure 3A). Ouabain was also unable to induce Ca^2+^ flashes in the presence of relatively selective Src family kinase inhibitor, PP2 (Bain et al., 2003) (Figure 3B). Furthermore, a specific inhibitor of the Na,K-ATPase-dependent Src kinase activation with pNaKtide (Li et al., 2009) also prevented the Ca^2+^ flashes induced by ouabain (Figure 3C).

### Microtubule network keeps the α2 isoform Na,K-ATPase and Src kinase together allowing the generation of Ca^2+^ flashes

**FIGURE 4:**
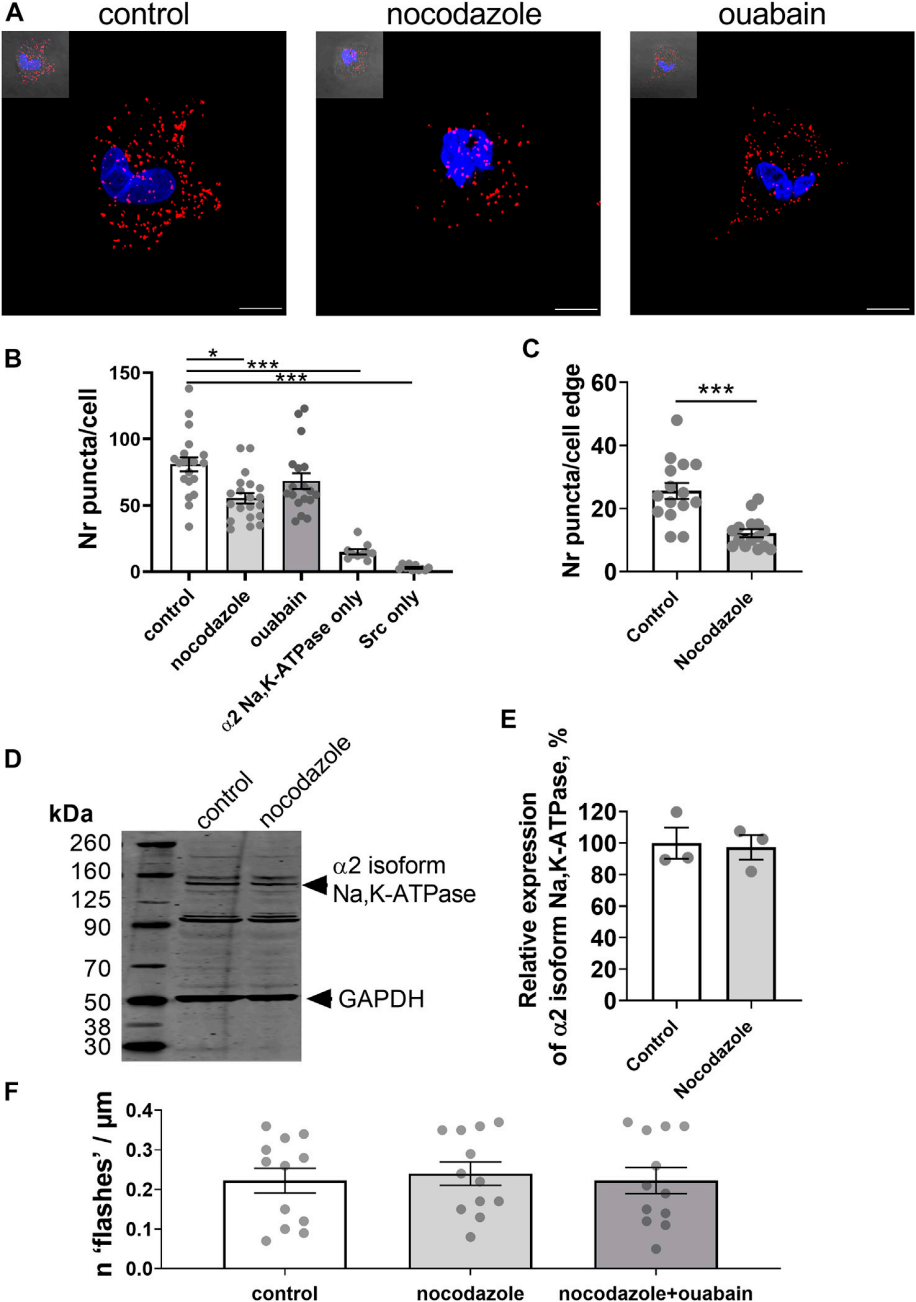
Microtubule network enables an interaction between the α2 isoform Na,K-ATPase and Src kinase, and the generation of Ca^2+^ flashes in A7r5 cells. Representative images of proximity ligation assay in A7r5 cells under control conditions or treated with nocodazole (10 µM) or with ouabain (10 µM) [**(A)**; scale bar = 10 µm]. Red punctae indicate a close (< 40 nm) proximity between the α2 isoform Na,K-ATPase and Src kinase. Nuclei are stained with DAPI and shown in blue. Quantification of the red punctae in the experiment as above [**(B)**; *n* = 18–20 of 4 cultures]. The red punctae were also counted only at the cellular edge **(C)** and nocodazole significantly decreased punctae in comparison with the control (*n* = 15 of 3 cell cultures). Representative Western blot detected the α2 isoform Na,K-ATPase in lysates of A7r5 cell under control conditions and treated with nocodazole **(D)**. GAPDH protein was stained for loading control. Quantification of the α2 isoform Na,K-ATPase expression under control conditions and after incubation with nocodazole [**(E)**; *n* = 3 of 3 cultures]. Nocodazole (10 µM) abolished the ouabain-induced Ca^2+^ flashes [**(F)**; *n* = 12 of 4 cultures]. * and ***, *p* < 0.05 and 0.001 (Kruskal–Wallis’ test followed by Dunn’s multiple comparison test in B, unpaired *t* test in C and E, and one-way ANOVA followed by Sidak’s multiple comparisons test in F).

An interaction between the Na,K-ATPase and tubulin, the main component of the microtubules, has previously been reported (Casale et al., 2003; Zampar et al., 2009). As the microtubule network is considered to play an important role in signal transduction (Gundersen and Cook, 1999), we hypothesized that it might be also involved in the generation of ouabain-induced Ca^2+^ flashes and tested this hypothesis in A7r5 cells (Figure 4A). We found a close proximity (< 40 nm) of the α2 isoform Na,K-ATPase and Src kinase (Figure 4B). Pre-incubation with nocodazole disrupted microtubules (Lindman et al., 2018) and reduced the number of co-localizations between the α2 isoform Na,K-ATPase and Src kinase compared to control, while ouabain treatment was without any effect (Figures 4A,B). When analysis was done only at the cell edge, the cells pre-treated with nocodazole had reduced co-localization the α2 isoform Na,K-ATPase and Src kinase in comparison with control cells (Figure 4C). This was not associated with changes in the α2 isoform Na,K-ATPase expression, which was similar in A7r5 cells under control conditions and after pre-treatment with nocodazole (Figures 4D,E).

To address the functional importance of an intact microtubule network for generation of ouabain-induced Ca^2+^ flashes, A7r5 cells were pre-incubated with nocodazole prior to stimulation with ouabain. Ouabain failed to induce Ca^2+^ flashes after disruption of microtubules with nocodazole (Figure 4F).

**FIGURE 5:**
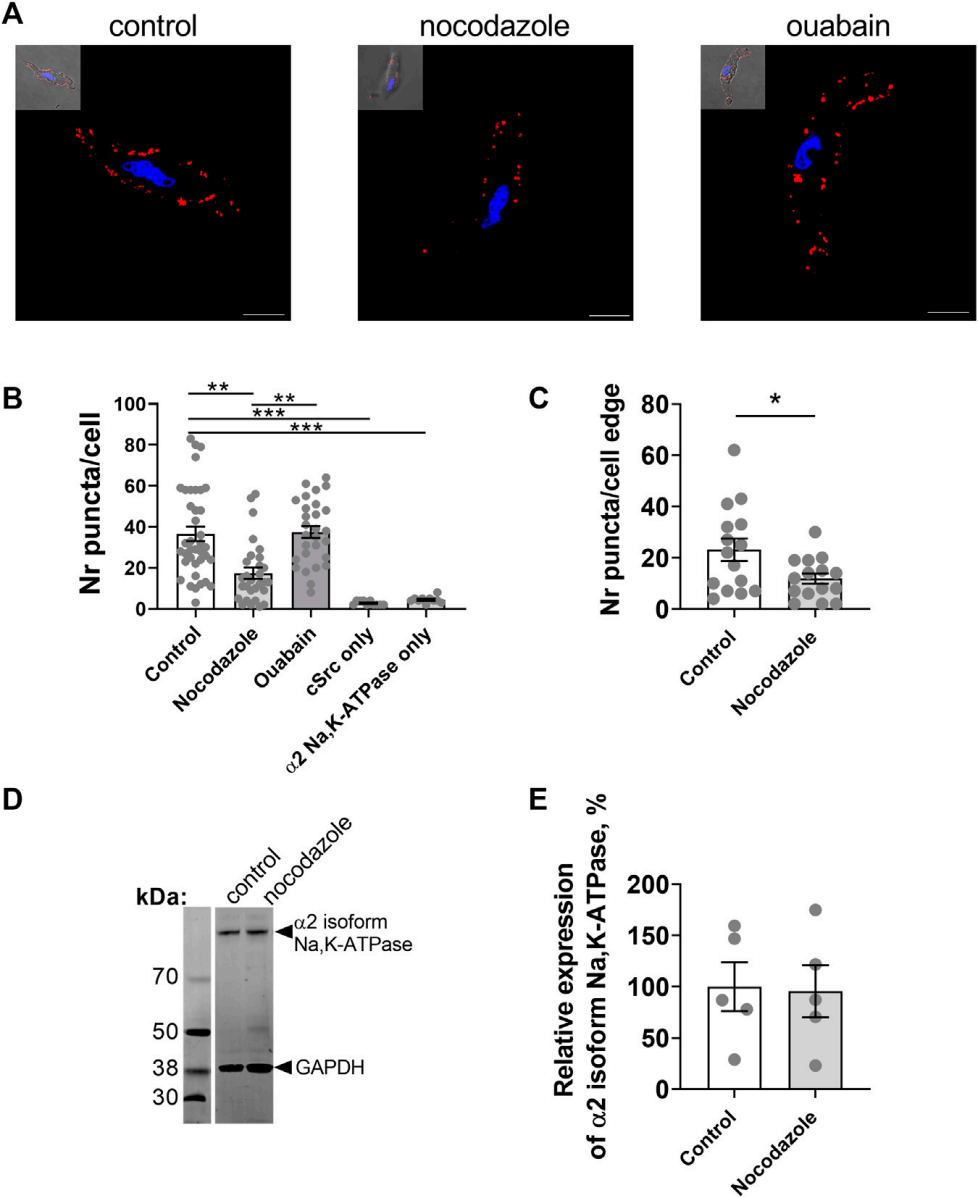
The interaction between the α2 isoform Na,K-ATPase and Src kinase in freshly isolated smooth muscle cells from rat mesenteric small artery is controlled by microtubule network. Representative images of proximity ligation assay in myocytes under control conditions and treated with either nocodazole (10 µM) or with ouabain (10 µM) [**(A)**; scale bar = 10 µm]. Red punctae indicate a close proximity (< 40 nm) between the α2 isoform Na,K-ATPase and Src kinase. Nuclei are stained with DAPI and shown in blue. Quantification of the red punctae in the experiment as in (A) is shown in [**(B)**; 10–37 cells from 3–4 rats]. At the cell edge nocodazole significantly decreased the red punctae in comparison with the control [**(C)**; 15 cells from 3 rats]. Representative Western blot with rat mesenteric arteries lysates for the α2 isoform Na,K-ATPase under control conditions and treated with nocodazole **(D)**. GAPDH protein was stained for loading control. Quantification of the α2 isoform Na,K-ATPase expression under control conditions and after incubation with nocodazole [**(E)**; *n* = 5]. *, ** and ***, denote *p* < 0.05, 0.01 and 0.001, respectively (Kruskal–Wallis’ test followed by Dunn’s multiple comparison test in B and unpaired *t* test in C and E (*p* = 0.90).

Experiments on freshly isolated rat mesenteric artery myocytes (Figure 5A) supported our findings from A7r5 cells (Figure 4). Nocodazole significantly decreased the number of co-localizations between the α2 isoform Na,K-ATPase and Src kinase in freshly isolated vascular smooth muscle cells (Figure 5B), but ouabain did not alter the number of interactions (Figure 5B). Nocodazole also decreased the co-localization at the cell edge (Figure 5C). Western blot analysis of rat mesenteric artery lysates showed that nocodazole did not change the total protein expression of the α2 isoform Na,K-ATPase (Figures 5D,E). Altogether, these results suggest that an intact microtubule network is required to maintain the α2 isoform Na,K-ATPase and Src kinase together allowing Src activation upon binding ouabain to the Na,K-ATPase.

### Nocodazole reduced Src phosphorylation and prevented ouabain induced Src phosphorylation

**FIGURE 6:**
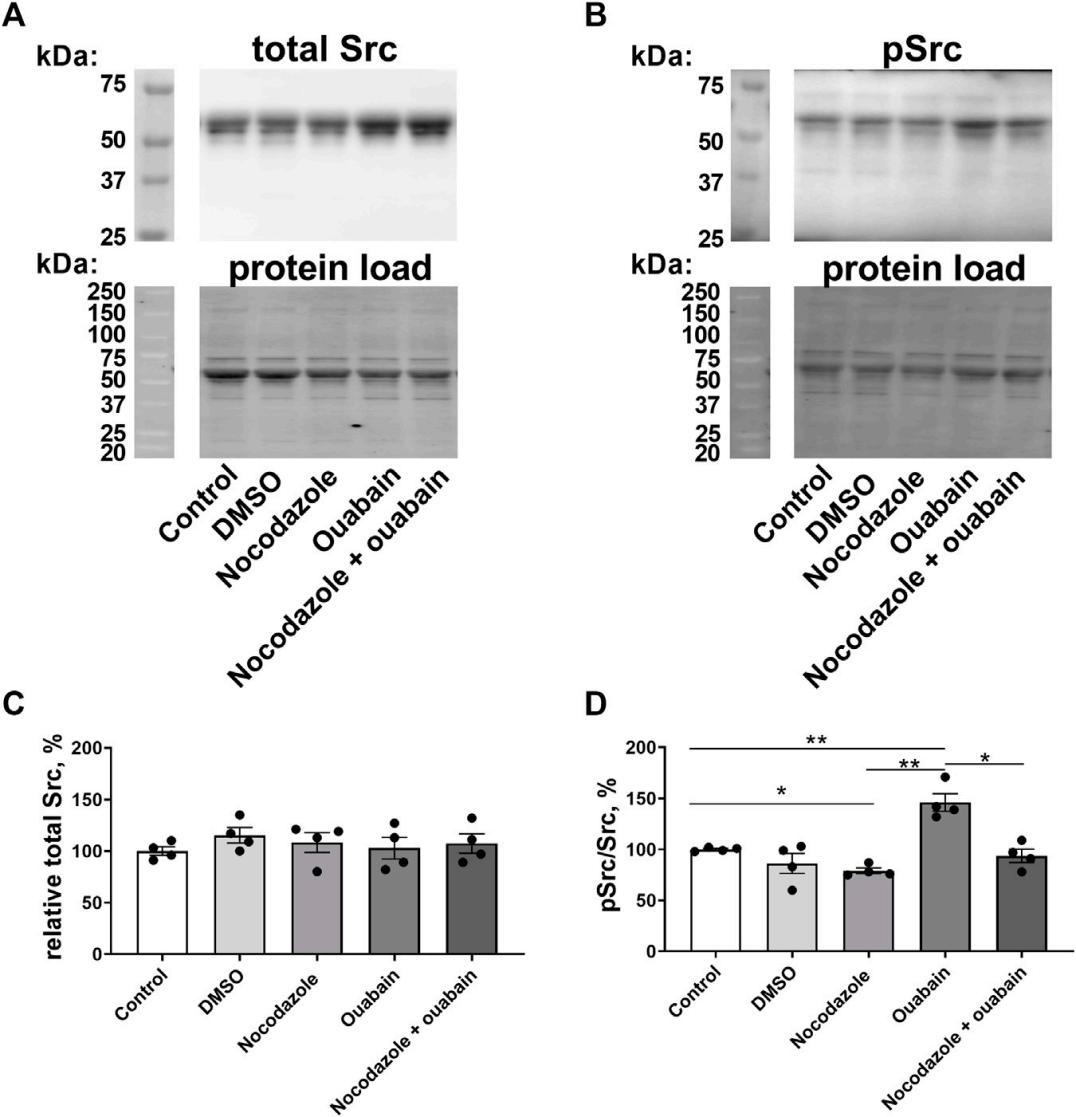
Ouabain increased Src kinase phosphorylation, while nocodazole prevented this increase. Representative blots from Western blot experiments where membranes were stained with either antibodies against total Src kinase **(A)** or with antibody against phosphorylated Src **(B)**. Lower panels in **(A,B)** show total protein load detected with stain-free membrane. Semi-quantification of total Src expression in A7r5 cells under control conditions, exposed to vehicle (DMSO), pre-incubated with nocodazole (10 µM), with ouabain (10 µM) or with a combination of nocodazole and ouabain **(C)**. Src phosphorylation relative to total Src content under control conditions, exposed to vehicle (DMSO), pre-incubated with nocodazole (10 µM), with ouabain (10 µM) or with a combination of nocodazole and ouabain **(D)**. *n* = 4, * and **, *p* < 0.05 and 0.01 (one-way ANOVA followed by Sidak’s multiple comparisons test in **(C,D)**.

Incubation with either nocodazole, ouabain, vehicle (DMSO), or with a combination of nocodazole and ouabain did not change the total Src content of A7r5 cells (Figures 6A,C). However, nocodazole reduced relative Src phosphorylation (Figures 6B,C). Moreover, although ouabain administration elevated relative Src phosphorylation, this was not seen in the presence of nocodazole (Figure 6C).

## Discussion

We showed previously that low concentrations of ouabain induced spatially restricted submembrane [Ca^2+^]_i_ transients in cultured aortic smooth muscle cells (Matchkov et al., 2007) and suggested the importance of these Ca^2+^ flashes for synchronization of contraction and intercellular coupling in the vascular wall (Matchkov et al., 2012; Hangaard et al., 2017). In the present study, we demonstrate that the generation of Ca^2+^ flashes requires the α2 isoform Na,K-ATPase, Src kinase and an intact microtubule network.

### The α2 isoform Na,K-ATPase-dependent activation of Src kinase is essential for generation of ouabain-induced Ca^2+^ flashes in smooth muscle cells

In this study, we show that Ca^2+^ flashes can be induced with 1 µM and higher concentrations of ouabain and suggest a primary role of Src activation for Ca^2+^ flash generation, although some modulatory role of membrane potential and trans-membrane ion translocation cannot be excluded completely. Accordingly, it has been suggested previously that ouabain mediates, at least in part, its pro-contractile and pro-hypertensive effects via ion transport independent signaling from the Na,K-ATPase Src activation (Yuan et al., 1993; Song et al., 2014).

Some controversy regarding the involvement of α1 and α2 isoforms of the Na,K-ATPase has been reported previously (Matchkov et al., 2012; Xie et al., 2015; Bouzinova et al., 2018; Staehr et al., 2018; Yu et al., 2018; Kutz et al., 2021). Although a difference in the studied tissues can contribute to the variability, previous studies demonstrated clearly that Src kinase has highest affinity to specific protein-domain on the α1 isoform (Tian et al., 2006; Li et al., 2009; Xie et al., 2015; Yu et al., 2018). However, we found in this study that a specific knock-down of the α2 isoform Na,K-ATPase abolished the ouabain-induced Ca^2+^ flashes in A7r5 cells. This cannot be because of associated changes in the expression of α1 isoform, as this siRNA knockdown protocol did not affect the expression of α1 isoform Na,K-ATPase, as we reported previously (Matchkov et al., 2012; Bouzinova et al., 2018). It has been shown previously that Src kinase binds to the Na,K-ATPase α1 isoform in E1 conformation (Ye et al., 2013). Changes in the activity of the α2 isoform Na,K-ATPase can modify localized concentration of Na^+^, which is important for E1/E2 conformation equilibrium of the α1 isoform (Jorgensen et al., 2003). However, an implication of both isoforms of the Na,K-ATPase-dependent in Src activation and Ca^2+^ flash generation is also possible. Although further analyses are needed, our results from proximity ligation assay demonstrated that the α2 isoform and Src kinase are localized in a proximity within 40 nm from each other. This is the first demonstration of an interaction between the α2 isoform and Src kinase in the cardiovascular system, although another study reported that both α1 and α2 isoforms interact with Src kinase in skeletal muscle cells (Kotova et al., 2006).

In this study, we inhibited the Na,K-ATPase-dependent Ca^2+^ flashes with two structurally unrelated tyrosine kinase inhibitors as well as with a specific inhibitor of ouabain-induced Src phosphorylation, pNaKtide (Li et al., 2009). This peptide was synthesized and validated for the specific inhibition of Src signaling from the α1 isoform Na,K-ATPase suggesting that this isoform is implicated in the signaling.

### The microtubule network is an important component of the Na,K-ATPase-dependent signaling

We found in this study that disruption of the microtubule network with nocodazole prevented the ouabain-induced Ca^2+^ flashes. The importance of the microtubule network for the enzymatic activity and signaling of the Na,K-ATPase has been suggested previously. That is, the interaction between the Na,K-ATPase and acetylated tubulin was shown in the rat brain (Alonso et al., 1998; Casale et al., 2003; Arce et al., 2008). Tubulin is the main component of microtubules and when acetylated, it can inhibit the enzymatic activity of Na,K-ATPase. Moreover, disruption of the microtubule network with nocodazole dissociated the complex between tubulin and Na,K-ATPase followed by increased activity in the Na,K-ATPase (Casale et al., 2005). Tubulin is able to interact in a direct manner through cytoplasmic domain 5 of the Na,K-ATPase, suggesting a role for the pump as a microtubule-plasma membrane anchorage site (Zampar et al., 2009; Santander et al., 2019). Mechanical response, i.e., membrane deformation and tension in response to osmotic stress, was suggested to regulate this interaction (Rivelli et al., 2012; Nigra et al., 2016). Moreover, the importance of the microtubule network for Src activation and trafficking was highlighted in different cell types (Abu-Amer et al., 1997; Yamada et al., 2000; Suter et al., 2004). Microtubules were also shown to play a key role in the regulation of steady-state Src activation at the plasma membrane (Wu et al., 2008).

In this study, we show a close proximity localization of the Na,K-ATPase α2 isoform and Src kinase, which was disturbed by nocodazole. Although we have not measured the expression of Src kinase in the presence of nocodazole, the unchanged expression of the Na,K-ATPase suggests that the reduced PLA signal after short incubation with nocodazole is a result of reduced interaction of these proteins. Accordingly, nocodazole decreased Src phosphorylation at resting conditions, and prevented its phosphorylation upon ouabain administration. It has been reported previously that the microtubule network is involved in Src vesicle trafficking, where nocodazole disrupted the trajectory of Src vesicles but did not inhibit their movement (Arnette et al., 2016). Surprisingly, administration of ouabain did not change proximity of the Na,K-ATPase α2 isoform and Src kinase. This may suggest that the proximity of these two proteins is important for initiation of intracellular signaling and Ca^2+^ flash generation but does not imply large intracellular movement of Src kinase. Although the importance of the microtubules for cell division, differentiation, motility, trafficking and intracellular signaling is accepted, very little is known about the role of microtubule network in vascular smooth muscle cells (Brozovich et al., 2016; Jepps, 2021). The importance of intact microtubule network for interaction between the Na,K-ATPase α2 isoform and Src kinase was validated in both cultured A7r5 cells and freshly isolated vascular smooth muscle cells suggesting the importance of this signaling downstream from the Na,K-ATPase for the regulation of vascular function *in vivo*. Based on this and previous studies on the Kv7.4 channel trafficking (Lindman et al., 2018; van der Horst et al., 2021), we suggest that the intact microtubule network in vascular smooth muscle cells is a key element to maintain vascular function.

### The functional implications of Ca^2+^ flashes

The transient changes in intracellular Ca^2+^ induced by micromolar ouabain were shown to be spatially restricted to submembrane regions, whereas no fluctuation in Ca^2+^ was seen in the center of the cells. In accordance with previous reports (Mulvany et al., 1984; Aalkjaer and Mulvany, 1985; Matchkov et al., 2007; Matchkov et al., 2012; Hangaard et al., 2017), ouabain did not induce global changes in intracellular Ca^2+^ concentration but elicited spatially restricted Ca^2+^ transients, i.e., Ca^2+^ flashes. Spatial and temporal changes in intracellular Ca^2+^ are critical for vascular smooth muscle cells where Ca^2+^ has been shown to act as a second messenger and initiate contraction (Wamhoff et al., 2006). Moreover, intracellular Ca^2+^ transients were shown to modulate a phenotype of vascular smooth muscle cells via regulation of transcription factors (Kudryavtseva et al., 2013). Although, functional significance of the ouabain-induced Ca^2+^ flashes was beyond the scope of this study (Glavind-Kristensen et al., 2004; Matchkov et al., 2007; Matchkov et al., 2012; Hangaard et al., 2015; Hangaard et al., 2017; Bouzinova et al., 2018; Staehr et al., 2018; Kutz et al., 2021), we can suggest based on previous reports that this spatial and temporal Ca^2+^ signaling could play an important role in vascular remodeling and contraction, intracellular signaling and smooth muscle cell metabolism. This study adds further complexity in the signaling by showing the key role of microtubule network in the Na,K-ATPase-dependent intracellular signaling mediated by Src kinase.

## Data Availability

The raw data supporting the conclusions of this article will be made available by the authors, without undue reservation.
